# The Effect of Recombinant Human Follicle-Stimulating Hormone on Sperm Quality, Chromatin Status and Clinical Outcomes of Infertile Oligozoospermic Men Candidate for Intracytoplasmic Sperm Injection: A Randomized Clinical Trial

**DOI:** 10.22074/IJFS.2021.6210

**Published:** 2021-01-27

**Authors:** Atefeh Verdi, Mohammad Hossein Nasr-Esfahani, Mohsen Forouzanfar, Marziyeh Tavalaee

**Affiliations:** 1 Department of Biology, Fars Science and Research Branch, Islamic Azad University, Fars, Iran; 2 Department of Biology, Shiraz Branch, Islamic Azad University, Shiraz, Iran; 3 Department of Reproductive Biotechnology, Reproductive Biomedicine Research Center, Royan Institute for Biotechnology, ACECR, Isfahan, Iran; 4 Isfahan Fertility and Infertility Center, Isfahan, Iran; 5 Department of Biology, Marvdasht Branch, Islamic Azad University, Marvdasht, Iran

**Keywords:** Follicle-Stimulating Hormone, Intracytoplasmic Sperm Injection, Male Infertility, Oligozoospermia

## Abstract

**Background:**

Follicle-stimulating hormone (FSH) plays a crucial role in spermatogenesis; in this study, we assessed the effect of recombinant human FSH (rhFSH) on sperm parameters, chromatin status and clinical outcomes of infer- tile oligozoospermic men candidates for intracytoplasmic sperm injection (ICSI).

**Materials and Methods:**

This interventional randomized clinical trials (IRCT) included 40 infertile oligozoospermic men undergoing ICSI. These individuals were randomized into two groups: 20 men received rhFSH drug for three months and the other 20 men who did not receive rhFSH drug were considered the control group. Before and 3 months after treatment initiation, sperm parameters (using computer-assisted semen analysis) and chromatin status [using chromomycin A3, aniline blue, and sperm chromatin dispersion (SCD) tests] were assessed in these individuals. Fur- thermore, hormonal profile was assessed using enzyme-linked immunosorbent assay (ELISA). Clinical outcomes of ICSI were also compared between the two groups.

**Results:**

The rhFSH treated group showed a significant increase in the level of FSH, luteinizing hormone (LH), tes- tosterone (T) and prolactin (PRL), as well as significant improvements in sperm parameters compared to the control group. Also, after administration of rhFSH, there was asignificant reduction in the percentage of sperm DNA damage, protamine deficiency and chromatin immaturity, while such a reduction in these parameters was not observed in the control group. Moreover, the percentage of embryos with grade Aquality, was significantly higher in the rhFSH group compared to the control group. The pregnancy rate in the rhFSH group was higher than the control group but the dif- ference was insignificant.

**Conclusion:**

Administration of rhFSH improves sperm quality in infertile oligozoospermic men and results in higher rates of good quality embryos post-ICSI (Registration number: IRCT20170923036334N2).

## Introduction

Reduced sperm count termed based on World Health Organization (WHO) criteria as “oligozoospermia”, is known as one of the major causes of male infertility and its prevalence shows regional variations (1). Commonly, this abnormality is accompanied by reduced percentage of sperm motility. Previous studies showed that oligozoospermia is a multifactorial condition in which genetic factors, such as chromosomal and single gene alterations, account for 20-30% of the cases (2, 3). In addition, other factors including hormonal imbalance, environmental factors, varicocele, sexually transmitted diseases, obstruction, testicular trauma, secondary testicular failure, infection and inflammation may be considered other etiological factors for the condition (3-5). Among the aforementioned etiologies, hormonal imbalance due to improper function of hypothalamicpituitary- gonadal (HPG) axis, is considered one of the main underlying reasons for reduced sperm production leading to oligozoospermia. In male, this axis controls sperm production and is governed by release of gonadotropinreleasing hormone (GnRH) from the hypothalamus to the anterior pituitary gland leading eventually to release of luteinizing hormone (LH) and follicle-stimulating hormone (FSH) which results in testicular production of estrogen and testosterone (T) hormone which are required for spermatogenesis (6-8). In this context, it was shown that aging- derived structural changes in median eminence, alter GnRH release and can affect FSH and LH production (9). Another cause of oligozoospermia is reduced FSH level in conditions such as Kallmann syndrome, isolated FSH deficiency or hyperprolactinemia. Moreover, environmental and lifestyle factors can also cause FSH reduction (10).

Considering the role of FSH in the process of spermatogenesis, numerous studies have evaluated the effect of FSH administration on improving spermatogenesis with the hope of increasing sperm count. The results of these studies have shown that gonadotropins in normal-low range, are not able to maintain normal spermatogenesis (14, 15) and in several studies, the effectiveness of FSH administration on improving sperm quality, was observed (16, 17). Also, it has been reported thatin both assisted reproductive technique and natural cycles, FSH administration for 3 months, improves pregnancy rate (16).

In this regard, Simoni et al. (18) showed that FSH administration in men with idiopathic infertility, not only improved sperm parameters, it also increased DNA integrity. Similarly, Colacurci et al. (16) demonstrated that after 3 months of FSH therapy, sperm DNA integrity was significantly improved in infertile men with idiopathic oligozoospermia. In addition, Kamischke et al. (19) showed that unlike sperm motility, volume of testis and sperm DNA condensation improved after treatment with daily 150 international units (IU) of rhFSH. Recently, Ding et al. (13) demonstrated that sperm parameters significantly improved after treatment with rhFSH in infertile men especially in individuals with normal- and low-level of inhibin B. Moreover, Paradisi et al. (20) in a pilot study, stated that a high-dose of rhFSH (daily 300 IU) can significantly increase sperm concentration and count, but could not significantly improve sperm motility or morphology.

FSH receptor defects are known as a potential risk factor for spermatogenetic failure. Selice et al. (21) assessed the effect of FSH treatment on individuals with polymorphism in FSH receptor gene and observed a significant improvement in sperm parameters in oligozoospermic men. In addition, a study by Italian Society of Andrology and Sexual Medicine showed that FSH therapy can improve both quantitative and qualitative sperm parameters and pregnancy rate in idiopathic oligoasthenoteratozoospermic men (22).

According to the above-mentioned statements, the aim of this study was to assess rhFSH therapy effects on sperm parameters, chromatin status, reproductive hormones, and clinical outcomes in oligozoospermic infertile men who were candidates for ICSI.

## Materials and Methods

### Study design

This randomized interventional clinical trials was reviewed and approved by the Ethics Committee of Islamic Azad University- Qom branch (IR.IAU.Qom. REC1396.56), and registered in Iranian registry for clinical trials (IRCT20170923036334N2). Initially, the aim, design, inclusion, and exclusion criteria of the study, were described to couples. 

Briefly, the couples were informed regarding the aim of this study; this study aimed at assessment of the effect of rhFSH treatment on sperm parameters, chromatin status and hormonal levels (as the primary aims) as well as clinical outcome of ICSI (as the secondary) in oligozoospermic individuals’ candidate for ICSI. The couples were informed that rhFSH therapy may improve semen quality and subsequently, the ICSI outcome. Men with varicocele or a history of varicocele surgery, systemic diseases, chemotherapy or radiotherapy history, or anatomical problems in genitals or those who were taking medications for systemic diseases or/and depression were not eligible to participate in this study. Therefore, the couples who accepted to participate in this study, were enrolled in to the “rhFSH/treatment group” and couples that refused to be treated with rhFSH but provided semen and blood samples for our study and allowed us to use their clinical data for this study, were considered the “control group”. 

The inclusion criteria were: low levels of FSH (less than 3 mIU/ml), age of 25-45 years, a history of infertility for at least 2 years, sperm concentration <15×10^6^ (i.e. oligozoospermia) according to WHO criteria (23). All the individuals conformed with the study and therefore, no couples were excluded from this study. The sample size was calculated based on the following equation:

n=(Z1-a2)2*(p*(1-p))(d)2

**Fig.1:**
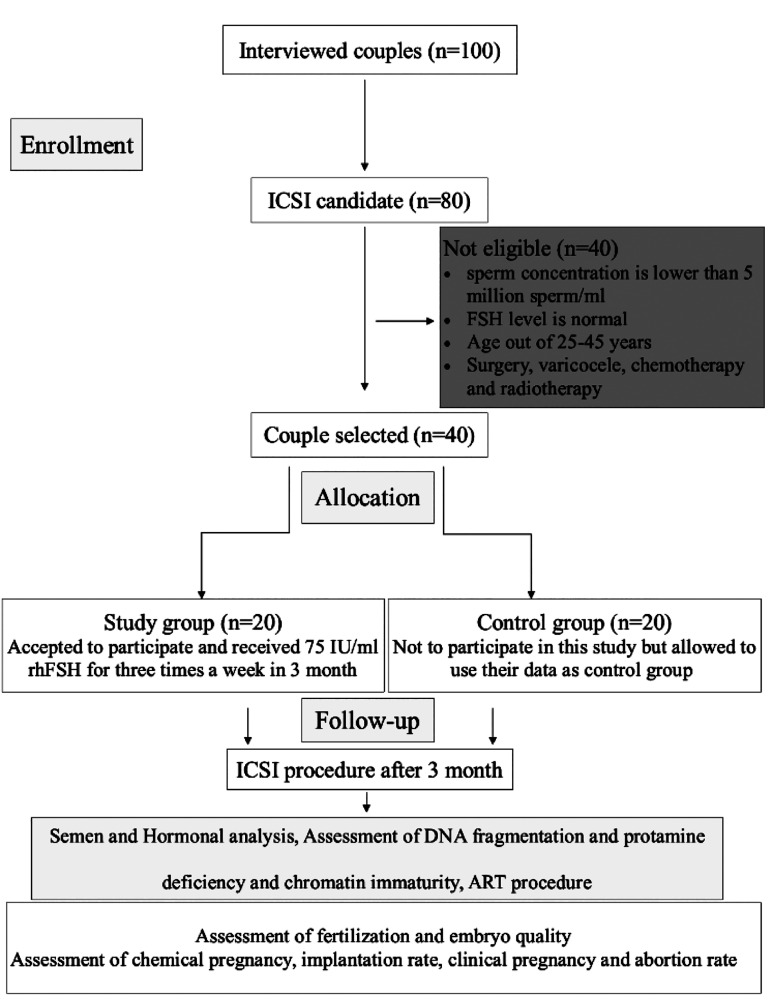
Flow diagram of study design. ICSI; Intracytoplasmic sperm injection and ART; Assisted reproductive technology.

Based on a 10% improvement for each parameter of the primary aims and a confidence interval of 1.96, and 0.1 value, a minimum of 20 individuals were calculated to be included in each group. Therefore, the first 20 individuals who accepted to receive 75 IU rhFSH (Gonal-F), three times a week for 3 months according to a previous study (24), were included in the rhFSH/treatment group, and the first 20 individuals who refused to be treated with rhFSH but provided semen and blood samples for our study and allowed us to use their clinical data for this study, were considered the “control group” (Fig .1).

### Semen analysis

Semen analysis was performed after liquefaction of samples obtained by masturbation after 3-5 days of sexual abstinence. Each parameters and the number of leucocytes were assessed respectively by CASA software and peroxidase staining according to WHO criteria (23).

### Hormonal analysis

After collection, blood samples were immediately centrifuged for 10 minutes at 3000 rpm (Hettich, EBA20, UK) and sera were separated and stored at -70^°^C. FSH, LH, PRL and total T levels were determined by ELISA method (Demeditec Diagnostics GmbH, Germany). LH and FSH are expressed as mIU/ml while total T and PRL are expressed as ng/ml.

### Assessment of DNA fragmentation

DNA fragmentation was assessed using an improved sperm chromatin dispersion (SCD) test (the Halosperm kit, INDAS laboratories, Spain). For each sample, a minimum number of 500 sperm was evaluated under the ×100 objective of an optical microscope. The following five patterns of halo around sperm head were detectable: i. Sperm with large halos (halo width of sperm equal to or higher than the minor diameter of the core), ii. Sperm with medium size halos (halo width of sperm between large and small halo), iii. Sperm with very small size halo (halo width of sperm smaller than one-third of the minor diameter of the core), iv. Sperm without a halo, and v. Degraded sperm. Sperm with small halos, without halos and degraded sperm were considered sperms with fragmented DNA, and data calculated for each sample was reported as percentage (25).

### Assessment of protamine deficiency and chromatin immaturity

For evaluation of protamine deficiency, initially, the semen sample was washed with PBS and then, fixed using methanol and glacial acetic acid (3:1 ratio) and stained with chromomycin A3 (CMA3, Sigma, USA) according to NasrEsfahani et al. (26). In addition, we assessed chromatin immaturity by aniline blue staining according to Terquem and Dadoune (27). The results are expressed as percentage of protamine deficiency and chromatin immaturity.

### Assisted reproductive technology procedure

For ovulation induction, the agonist protocol was used. On day 2 of the cycle, a vaginal ultrasound was carried out to confirm absence of any active follicle and presence of thin endometrium. Following confirmation of basal characteristics, gonadotrophins were administered at 150- 225 IU/ml daily. A second ultrasound was carried out on day 6 to monitor follicular growth and adjust drug dose. Suppression using a GnRH antagonist started on day 7 when leading follicles were around 12-14 mm. Triggering was done by 10000 IU/ml human chorionic gonadotropin (hCG) when 3 mature leading follicles were greater than 17 mm.

Thirty-six hour post triggering, oocytes were recovered by transvaginal ultrasound and then, the standard ICSI protocol was performed.

At 16-18 hours after ICSI, fertilization was confirmed by the presence of two pronuclei (2PN) and rate of fertilization was calculated as follows: the number of 2PNs divided by the number of MII oocytes in seminated by ICSI procedure. Three-day post ICSI, embryo quality was assessed based on a three-point scoring system (28, 29): i. Absence or fragmentation of <25% on embryonic surface, ii. Equality of blastomere’s size and shape, and iii. Blastomere cell number greater or less than 7. Embryos presenting all above-mentioned criteria were scored as “A”, embryos having only two criteria were scored as “B” and embryos presenting only one criteria were scored as “C”. All three group (A+B+C) were chosen and the percentage of top-quality embryos was calculated.. The embryos were selected for transfer based on availability, with the following priority: A, B and finally, C. All patients received progesterone supplementation as luteal phase support. Two weeks after embryo-transfer, chemical pregnancy was confirmed by assessment of serum β-hCG. Clinical pregnancy was diagnosed by ultrasonographic visualization of one or more gestational sacs (30).

### Statistical analysis

Statistical analysis was performed using SPSS version 21.0 (SPSS Inc. Chicago, IL). For comparison of study parameters between the control and rhFSH groups before and after 3 months, paired sample t test was used. Chisquare was used for comparison of pregnancy outcomes and implantation rate between the two groups. P≤0.05 were considered significantly different.

## Results

In this study, the average age of oligozoospermic men was 33.85 ± 4.5 years old in the rhFSH group and 36.25 ± 5.22 years old in the control group with no significant differences between the two groups. The mean of body mass index (BMI) showed no significant difference in rhFSH (28.8 ± 1.8 vs. 29.09 ± 1.8, respectively, P=0.498) or control groups (29.36 ± 1.79 vs. 29.33 ± 1.86, respectively, P=0.683) when comparing before treatment and after 3-month treatment values. The comparison of before treatment and after 3-month treatment values in terms of sperm function and hormonal parameters in the rhFSH and control groups, is shown in Table 1 and Figure 1. Unlike the control group, all the sperm parameters (sperm count, concentration, motility, and morphology), chromatin status (sperm DNA fragmentation, protamine deficiency, and chromatin immaturity) and hormonal profile (FSH, LH, T and PRL) significantly improved after treatment of oligozoospermic men with rhFSH (P<0.05).

In addition, we followed up the clinical outcomes of oligozoospermic men in both groups and the results are presented in Table 2. Males and females age and the number of oocytes retrieved were not significantly different between the two groups (P=0.13), while the mean number of MII oocytes (P=0.02) and embryo transfer (P=0.02) were significantly higher in the rhFSH group compared to the control group. We found that the mean percentage of fertilization rate, embryos with grade B quality and chemical and clinical pregnancy rates were higher in the rhFSH group than the control couple, but differences were not significant. Only, the mean percentage of embryos with grade A quality was significantly higher in the rhFSH group compared to the control group (P=0.026).

**Table 1:** Comparison of sperm parameters and hormonal profile before and after 3 months in the rhFSH and control group


Parameters	rhFSH n=20		Control group n=20	
	Before	After	Before	After

Sperm concentration (×10^6^/ml)	7.6 ± 2.5	18 ± 3.5^a^	7.9 ± 3.9	8.03 ± 3.8
Total sperm count (×10^6^/ejaculate)	26.9 ± 17.6	65.2 ± 28.9^a^	13.4 ± 7.1	14.2 ± 7.1
Total sperm motility (%)	19.75 ± 10.7	33.5 ± 14.1^a^	14.25 ± 6.9	14 ± 6.2
Progressive sperm motility (%)	4.5 ± 5.3	17.5 ± 10.4^a^	6.5 ± 2.3	6.8 ± 2.05
Immotile sperm (%)	80.25 ± 10.7	66.5 ± 14.15^a^	85.75 ± 6.9	86.25 ± 6.4
Sperm abnormal morphology (%)	98.7 ± 0.5	97 ± 0.8^a^	99 ± 0.00	98.85 ± 0.4
FSH (mIU/mL)	1.83 ± 0.5	3.88 ± 0.9^a^	1.76 ± 0.3	1.79 ± 0.3
LH (mIU/mL)	4.99 ± 1.9	6.048 ± 2.1^b^	5.03 ± 1.7	5.06 ± 1.6
Testosterone (ng/mL)	3.034 ± 1.03	4.56 ± 1.1^a^	3.5 ± 1.05	3.55 ± 1.0
Prolactin (ng/mL)	10.56 ± 2.5	12.69 ± 2.7^a^	9.69 ± 3.6	9.58 ± 3.5


Data are presented as means ± SD. ^a^ ; P<0.001, ^b^; P<0.05 indicate significant differences when comparing before and after treatment values. Paired sample t test was used for comparison of parameters before treatment and after three-month treatment. rhFSH; Recombinant human follicle-stimulating hormone, and LH; Luteinizing hormone.

**Fig.2:**
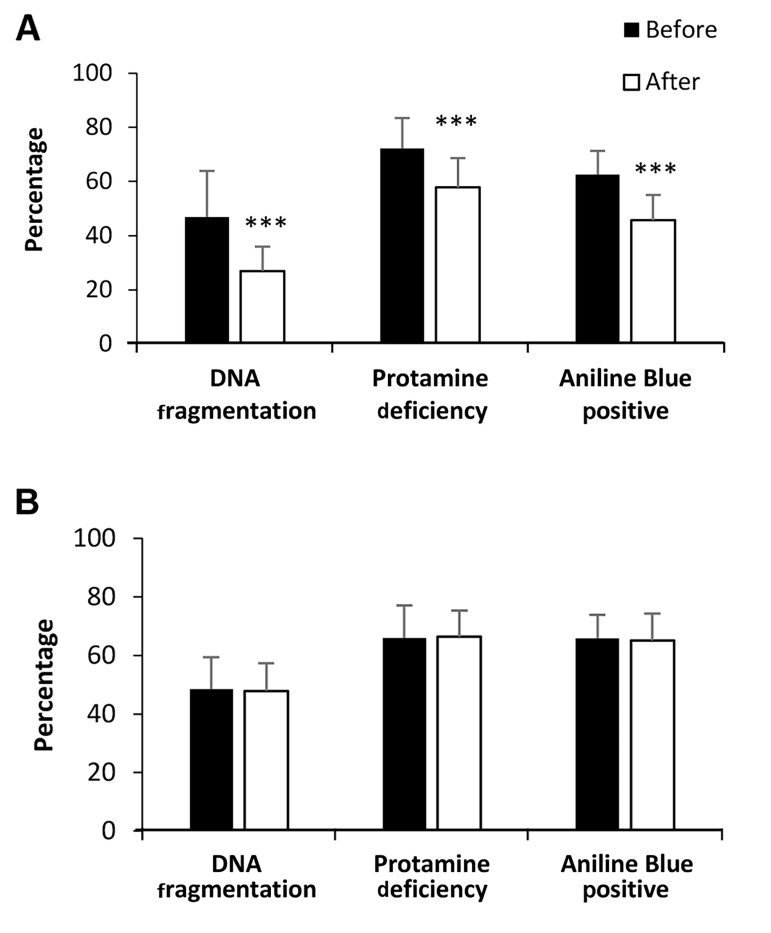
Sperm genomic content comparison. Comparison of mean percentage of sperm DNA fragmentation, protamine deficiency, and chromatin immaturity before treatment and after three-month treatment in the **A.** rhFSH and **B.** Control groups. Paired sample t test was used for comparison of parameters between the two groups. All data are presented as mean ± SD. ***; Show significant differences at P<0.001 and rhFSH; Recombinant human follicle-stimulating hormone.

**Table 2:** The comparison of, males and females age, the number of oocytes, and ICSI clinical outcomes between the rhFSH and control groups


Parameters	rhFSH group	Control group	P value

Male age (Y)	33.85 ± 4.5	36.25 ± 5.22	0.13
Female age (Y)	28.8 ± 4.9	31.65 ± 4.72	0.07
Number of oocytes	10.55 ± 5.763	7.05 ± 4.123	0.33
Number of MII oocytes	7.5 ± 4.007	4.9 ± 2.8	0.02
Number of embryo transfers	2.06 ± 0.8	1.137 ± 1.8	0.02
Fertilization rate (%)	65.8	59.7	0.56
Embryo quality grade A (%)	16.6	9.7	0.026
Embryo quality grade B (%)	73.4	64.5	0.69
Embryo quality grade C (%)	10	25.8	0.85
Biochemical pregnancy rate (%)	50	25	0.13
Clinical pregnancy rate (%)	35	20	0.48


Data are presented as mean ± SD or percentage. ICSI; Intracytoplasmic sperm injection and rhFSH; Recombinant human follicle-stimulating hormone. Independent samples t test was used for comparison of these parameters between the two groups, except for pregnancy rate compared by Chi-square test.

## Discussion

The results of the current study clearly showed that mean percentage of sperm parameters, protamine deficiency, DNA fragmentation, and chromatin immaturity were significantly improved in oligozoospermic men treated with rhFSH for three months compared to untreated oligozoospermic men. In addition, hormonal profile of these individuals was significantly improved compared to the control group. These results showed that administration of rhFSH was effective in improving spermatogenesis function in oligozoospermic men. In this regard, several clinical trials, and a study in a monkey model revealed an increase in testicular volume after FSH treatment, indicating that FSH hormone could increase germ cell proliferation in seminiferous tubules (19, 31, 32). In the light of these considerations, we assessed the effect of rhFSH on sperm functional parameters and clinical outcomes in infertile oligozoospermic men candidate for ICSI.

With regard to sperm DNA fragmentation, Ruvolo et al. (17) showed that FSH administration improves sperm DNA damage in men with hypogonadotropic hypogonadism and idiopathic oligozoospermia with high DNA fragmentation. Unlike the results of current study, others (19) did not observe any improvement in sperm parameters following administration of rhFSH in infertile men. The only parameters that was improved in foregoing study were testicular volume and sperm DNA fragmentation compared to the placebo group. The difference in the results of Kamischke et al. (19) study and our study could be related to the type of selected patients as we only focused on oligozoospermic individuals while they included cases with previous failed fertilization, azoospermic individuals undergoing testicular biopsies,* in vitro* fertilization (IVF) and ICSI.

In addition to sperm functional parameters, in the current study, clinical outcomes of ICSI in both groups were assessed and it was interesting that mean percentage of good quality embryos was significantly higher in the rhFSH compared to the control group. Although, the mean values for percentages of clinical and chemical pregnancy improved in the rhFSH compared to the control group, but the difference was not significant. Considering the impact of DNA fragmentation on development, several studies have shown associations between sperm DNA fragmentation and early embryonic development. But the association between fertilization rate and sperm DNA fragmentation remains controversial (31-34). Indeed, some authors have shown that even treatment of sperm with H_2_O_2_ does not preclude pronuclei formation (35). Most researchers believed that, due to DNA repairing mechanism in oocyte and early embryos, the effect of DNA damage on development should be observed after maternal-zygotes genomic transition which takes place at around 6-8-cell stage in human (36). Therefore, some studies have observed a significant effect for sperm DNA fragmentation on the quality of embryos on day 3 (32) while others believed that the effect of sperm DNA fragmentation shall be observable at around blastocyst stage when zygotic genome activation is more complete (37-39). In this study, since the common day for embryo transfer is day 3, we could not assess the effect of sperm DNA fragmentation on blastocyst formation rate, but we observed a significant improvement of sperm DNA fragmentation after rhFSH therapy, and a significant rate of good quality embryos on day 3 which is consistent with previous studied in this filed (31, 32).

If sperm DNA fragmentation is not repaired by oocyte or embryo, the embryos may have a reduced chance of implantation and inducing a pregnancy. In this study, we also showed that rhFSH therapy insignificantly improved the pregnancy rate. Although an improvement in pregnancy rate was expected, but not asignificant improvement was observed in this respect that is probably due to small sample size of this study. In addition, the mean number of MII oocytes was significantly higher in the rhFSH group compared to the control group. Obviously, this wasnot related to rhFSH in male and is regarded as a “bias” in the current results which is likely related to sample size and could doubtlessly impact the clinical outcomes of these couples. Therefore, further studies are needed to assess the effect of rhFSH therapy on clinical outcomes in oligozoospermic men in large populations. On the other hand, we highlighted that therapy with rhFSH in infertile men with oligozoospermia was effective in terms of spermatogenesis level, sperm function parameters, and hormonal profile. According to the literature, administration of rhFSH in idiopathic male infertility, could improve spermatogenesis thought he eventual benefits of supra-physiological FSH on spermatogenesis remain unclear. In this regard, Santi et al. (40) stated that the supporting effect of rhFSH on spermatogenesis should be assessed throughevaluation of sperm parameters as the primary endpoint. Therefore, our results confirm this theory.

## Conclusion

Taken together, based on the results of this study, treatment of idiopathic oligozoospermic individuals with rhFSH not only improves sperm parameters sperm chromatin integrity and hormonal profile, but also significantly improves embryo quality post ICSI and insignificantly improves the pregnancy rate. 
